# Combined Analytical Approaches to Standardize and Characterize Biomaterials Formulations: Application to Chitosan-Gelatin Cross-Linked Hydrogels

**DOI:** 10.3390/biom11050683

**Published:** 2021-05-01

**Authors:** Sofia Magli, Lorenzo Rossi, Cesare Consentino, Sabrina Bertini, Francesco Nicotra, Laura Russo

**Affiliations:** 1 Department of Biotechnology and Biosciences, University of Milano-Bicocca, 20126 Milan, Italy; s.magli1@campus.unimib.it (S.M.); l.rossi58@campus.unimib.it (L.R.); francesco.nicotra@unimib.it (F.N.); 2 BioNanoMedicine Center, University of Milano-Bicocca, 20126 Milan, Italy; 3 G. Ronzoni Institute for Chemical and Biochemical Research, 20126 Milan, Italy; cosentino@ronzoni.it (C.C.); bertini@ronzoni.it (S.B.); 4 CÚRAM SFI Research Centre for Medical Devices, National University of Ireland Galway, Galway, Ireland

**Keywords:** hybrid biomaterials, gelatin, chitosan, Diels Alder, hydrogels, extracellular matrix

## Abstract

A protocol based on the combination of different analytical methodologies is proposed to standardize the experimental conditions for reproducible formulations of hybrid hydrogels. The final hybrid material, based on the combination of gelatin and chitosan functionalized with methylfuran and cross-linked with 4-arm-PEG-maleimide, is able to mimic role, dynamism, and structural complexity of the extracellular matrix. Physical–chemical properties of starting polymers and finals constructs were characterized exploiting the combination of HP-SEC-TDA, UV, FT-IR, NMR, and TGA.

## 1. Introduction

The design of new advanced hybrid hydrogels for tissue engineering is today a matter of great interest for a plethora of applications [1,2,3,4,5,6]. The advancement in fabrication methodologies, for instance, 3D printing and bioprinting, requires an increased knowledge of the molecular composition of the employed biopolymers, guaranteeing an improved control of their effect on extracellular matrix (ECM) signaling and remodeling [7,8,9,10]. The combination of different classes of biopolymers, such as proteins and polysaccharides, characterized by different impacts on cells viability and behavior, is a promising approach to build up structures mimicking the complex ECM architectures and role [11,12,13,14]. To accurately reproduce the ECM properties, new hybrid biomaterials made of natural and/or synthetic polymers are usually designed and synthesized by covalent cross-linking, taking advantage of functional groups already present in the polymer chains or properly introduced [15,16,17]. Furthermore, the combination of different polymers increases the system complexity, providing additional motifs for fine control over crucial features for the desired applications [18]. Thus, the physical features, 3D architecture, stiffness properties, and biochemical composition can be designed in a customizable way to strategically promote specific cues finely tuning cell behavior [19,20]. However, the employment of heterogeneous polymers combined with the assessment of new cross-linking methodologies embraces some limitations in standardization of the ultimate constructs. In order to overcome this limitation, a control over reaction kinetics is required, besides a deep characterization of the final product, performed by combining multiple techniques. 

The cross-linking method is, for multiple reasons, a crucial aspect to be assessed. For such purpose, several click reactions have been investigated, exploiting linkers of different length bearing functional groups complementary to those already present in polymers backbone or previously chemically introduced [14,21,22,23,24]. Among the established approaches, Diels Alder reaction has already been widely used with a significant assortment of biopolymers to control cross-linking and conjugations between polymers displaying diene and dienophile moieties [25,26,27,28,29,30,31,32,33,34]. However, the reported studies are often focused on homopolymer formulations [32,35,36,37]. In our group, a hybrid hydrogel was developed employing gelatin and chitosan functionalized with methylfuran and then cross-linking them by Diels Alder reaction with 4-arm-PEG10K-maleimide [15,34]. The obtained hydrogel (GelChiDA) showed stability over time in cell culture conditions, in both 3D scaffolds and 3D-bioprinted cell-embedded formulations. The development of such promising hydrogel requires, however, standardization of the process in order to guarantee reproducibility of homogeneous batches, which is critical for the translation to frontier research. Well-assessed protocols are needed to monitor the kinetics of the reactions, the structural features, and the characterization of the produced hydrogel after cross-linking reaction. Considering the complexity of the system, the reaction kinetics and physical–chemical properties are arduous to investigate. Here, we report the combination of different analytical methodologies, to follow the kinetics of GelChiDA formation and to characterize the final construct in terms of physical–chemical properties. To this purpose, we exploited the combination of HP-SEC-TDA, UV, FT-IR, NMR, and thermogravimetric analysis. 

## 2. Materials and Methods

Water-soluble chitosan was purchased from Biosynth Carbosynth (Compton, UK). All other chemicals were purchased from Merck KGaA (Darmstadt, Germany), unless differently stated, and used as received without further purifications. Deionized water (conductivity less than 0.1 S) was prepared with an inverse osmosis system (Culligan, Milan, Italy). PolyCAL TM Pullulan std-57k was used (Malvern Instruments Ltd., Malvern, UK). The reagent grades were 98%.

### 2.1. Sample Preparations

Functionalization of Gelatin and Chitosan with methylfuran. Gelatin and chitosan were functionalized with methylfuran as already reported [15]. Briefly, both polymers were reacted with 5-methylfurfural and NaBH_3_CN performing a reductive amination. The functionalized polymers (polymer-MFs) were dialyzed in ionic conditions and against H_2_O.

General procedure for hydrogel formation. The obtained polymer-MFs were freeze dried and employed for the hydrogel formation: 33 mg of gelatin-MF and 17 mg of chitosan-MF were dissolved in 1 mL of PBS pH 7.4 at 310 K. After total dissolution of polymers, 2.6 mg of 4-arm-PEG10K-maleimide was added, and the solution was stirred until its dissolution and then left at 310 K for 3 h allowing the hydrogel formation.

### 2.2. Analytical Methods

Molecular weight distribution by size exclusion chromatography with triple detector array (HP-SEC-TDA) analysis. Evaluation of the molecular weight distribution of gelatin and gelatin-MF samples was performed by size exclusion chromatography coupled with a multi-detector system (refractive index, right and low angle light scattering, viscometer, Viscotek mod. 305 Triple Detector Array, Malvern Instruments Ltd., Malvern, UK) [38]. In particular, the analysis of gelatin was performed at 313 K, using a TSKPWXL column (Tosoh Bioscience, 7.8 mm ID × 30 cm). A total of 0.01 M K buffer + 0.125 M NaNO_3_, containing 0.05% NaN3, pre-filtered using 0.22 mm filter (Millipore), was used as mobile phase at a flow rate of 0.6 mL/min. Briefly, 0.1 mL of sample were injected at about 1 mg/mL. Chromatographic conditions for gelatin-MF were set up using two TSKPWXL columns in series (Tosoh Bioscience, 7.8 mm ID × 30 cm, V_0_ 6 mL, Vt 11 mL each one) with an aqueous solution of 0.2 M NaNO_3_ added with 0.05% NaN_3_ pre-filtered onto 0.22 mm filter (Millipore), used as mobile phase at a flow rate of 0.6 mL/min. Then, 0.1 mL of each sample was injected at about 1 mg/mL. For both methods, columns, injector, and detectors were maintained at 313 K. For chitosan and chitosan-MF samples, chromatographic conditions were set up using two gel TSKWXL mixed bed columns (Tosoh Bioscience, 7.8 mm ID × 30 cm, V_0_ 6 mL, Vt 11 mL) with an aqueous solution of 0.3 M sodium acetate and 0.3 M acetic acid (pH 4) added with 0.05% NaN_3_ pre-filtered onto 0.22 mm filter (Millipore) used as mobile phase at a flow rate of 0.6 mL/min. Further, 0.1 mL of each sample were injected at about 1 mg/mL. Columns, injector, and detectors were maintained at 313 K. The detector was calibrated with the Pollulan narrow standard of known Mw, polydispersity, and intrinsic viscosity (Malvern Instruments Ltd., Malvern, UK). The chromatogram elaboration was performed using OmniSEC software version 4.6.2. For calculations, a differential refractive index increment (dn/dc) value of 0.180 for gelatin and its derivates and of 0.167 for chitosan and its derivates were used for converting RI voltages to solute concentration at each data slice across a chromatographic peak, as previously described [38,39,40]. The obtained Mw ad Mn values were rounded to the nearest 1000 Da. 

FTIR-ATR. Each sample was reduced as powders and FT-IR spectra were recorded in attenuated total reflection ATR mode using PerkinElmer Spectrum 100 FTIR Spectrometer (Milan, Italy). The absorption spectral range was collected between 4000 and 650 cm^−1^ at a spectral resolution of 2 cm^−1^ and 40 scans. FT-IR analysis was performed at different time points on freeze-dried samples.

UV−VIS Measures. The UV–VIS absorption-solution spectra of the reaction was followed in real time from T0 to 3 h. The spectra were recorded in the spectral region of 200–500 nm using JASCO V-770 spectrophotometer (Cremella, Italy). Briefly, 1 mL of the hybrid GelChiDA starting solution was transferred into a 0.1 mm optical path quartz cuvette, and the spectra were acquired between T0 and 3 h.

Procedure for hydrogel formation for ^1^H-NMR and Diffusion Order Spectroscopy NMR analysis. Gelatin-MF (33 mg) and chitosan-MF (17 mg) were dissolved in 1 mL deuterated PBS (dPBS) pH 7.4 at 310 K and vortexed to help the solubilization. Once a solution was obtained, 2.6 mg of 4-arm-PEG10K-maleimide was added and the sample was immediately transferred in an NMR tube and the analysis was started.

^1^H NMR kinetics experiments. ^1^H-NMR spectra were acquired using a Varian 400 MHz Mercury instrument (Palo Alto, Santa Clara, CA, USA), operating at a proton frequency equal to 400 MHz at 310 K. Chemical shifts were referenced towards 3-(trimethylsilyl)propionic-2,2,3,3-d_4_ acid sodium salt (0 ppm). Spectra of polymers were acquired setting the pulse angle at 90° and the relaxion delay at 2 s, while the number of scans varied between 160 and 240 depending on the signal-to-noise ratio. The hydrogel kinetics of gelatin with chitosan was monitored by the acquisition of ^1^H-NMR spectra over time. ^1^H-NMR spectra were acquired using 16 scans, with a pulse angle of 90° and relaxation delay of 20 s. Every 10 min, a spectrum was recorded for a total of 32 spectra. All NMR spectra were acquired in deuterated PBS, pH 7.4, containing 0.05% wt of 3-(trimethylsilyl)propionic-2,2,3,3-d_4_ acid sodium salt.

Diffusion Order Spectroscopy NMR (DOSY). DOSY experiments of both the hydrogel and the blended polymers were performed with Bruker ADVANCE NEO, 500 MHz spectrometer (Bruker, Karlsruhe, Germany) equipped with a 5 mm TCI cryo-genic probe, at 310 K, and measured using the 2D stimulated echo sequence using bipolar gradient pulse for diffusion. The hydrogel has been prepared as reported in material and method section, whereas the blended polymers were prepared in identical concentrations and conditions but without the 4-arm-PEG10K-maleimide, in ^1^H dimension of the diffusion experiments, a sweep width of 20 ppm was used. The gradient pulse (δ) was set to 5 ms, and the diffusion time (Δ) was set to 300 ms. The 2D DOSY experiments were acquired using the 2D-stimulated echo sequence with bi-polar gradient pulse for diffusion with gradients varying linearly from 5% to 95% in 32 steps with 16 scans per step. The gradient pulse (δ) and the diffusion time (Δ) were set to 5 and 300 ms, respectively. Spectra were processed by Top-Spin 4.0.6 software, supplied by the manufacturer. A 1-Hz line broadening Lorenzian function was applied, and each row was phased and baseline was corrected before Fourier transformation in the F2 dimension. The diffusion coefficients D for the 1H dimension in which molecules with different diffusion coefficients correlate to two distinct signals were calculated with Bruker Dynamic Center 2.5 (Bruker, Karlsruhe, Germany). The fitted function used is $I={I_{0}}^{\left(-D\cdot x^{2}\cdot \gamma^{2}\cdot \delta^{2}\right)\cdot \left(\Delta -\frac{\delta }{3}\right)}$ where *I* is the observed intensity, *I*_0_ is the reference intensity, *x* is the gradient strength, *D* is the diffusion coefficient, *γ* is the gyromagnetic ratio of the observed nucleus, *δ* is the length of the gradient, and Δ is the diffusion time.

*TGA analysis.* TGA of the starch–gelatin samples was performed using a Mettler Toledo TGA/DSC1 Star-e System. Samples of ~10 mg were subjected to temperatures ranging from 303 to 1073 K at a heating rate of 10 K min^−1^. Nitrogen was used as purge gas at a flow rate of 50 mL min^−1^. Samples were freeze dried prior detection.

## 3. Results

**Scheme 1:**
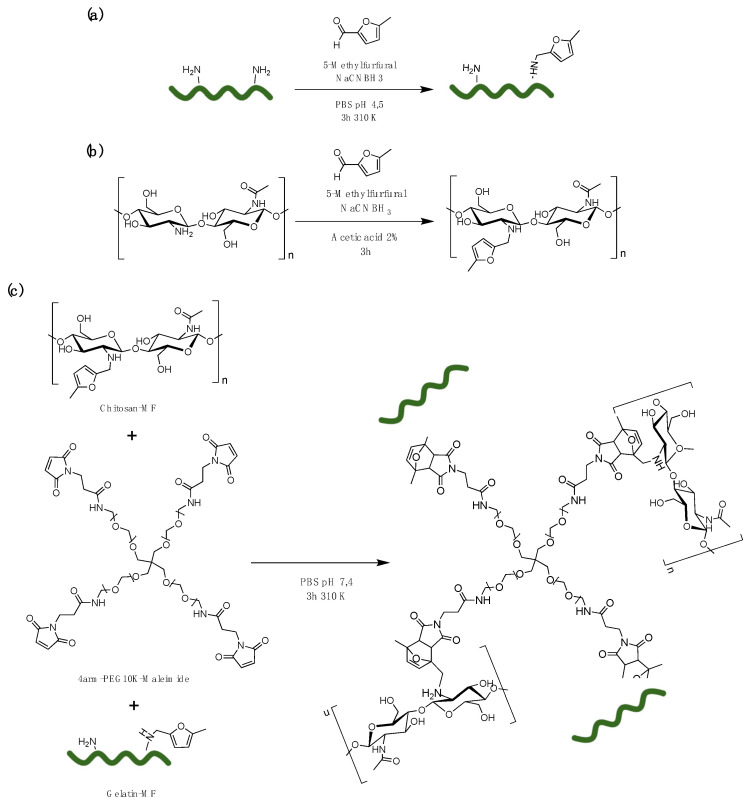
Synthetic scheme of chitosan (**a**) and gelatin (**b**) functionalization with 5-methylfuran and GelChiDA hybrid biopolymer cross-linking reaction (**c**).

Type A gelatin from skin porcine and water-soluble chitosan were employed as starting biopolymers [41,42,43,44]. These biopolymers were previously investigated in our group for the development of 3D bioprintable GelChiDA hydrogels for 3D cell cultures and tissue engineering purposes [15]. Briefly, both gelatin and chitosan were functionalized with methylfuran (MF), exploiting, respectively, the amino group of lysine residues of gelatin and the amino group of chitosan by reductive amination. The introduced dienes subsequently undergo cross-linking exploiting a Diels-Alder cycloaddition reaction using as dienophile a commercially available 4-arm-PEG10K-maleimide (Scheme 1), to obtain a heteropolymers based hydrogels [15]. As reported in a previous work, this cross-linking approach resulted in a highly reproducible, biocompatible, affordable, and easy to characterize product.

### 3.1. Molecular Weight Distribution by Size Exclusion Chromatography with Triple Detector Array (HP-SEC-TDA) Analysis

**Figure 1:**
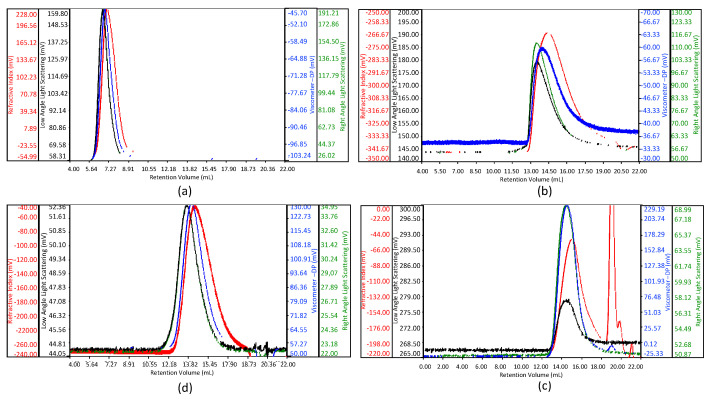
High-performance size exclusion chromatography on line with a triple detector array (HP-SEC-TDA) chromatogram of gelatin (**a**) and gelatin-methylfuran (MF) (**b**) chitosan (**c**) chitosan-MF (**d**) samples (red—refractive index; blue—viscometer; green—right-angle laser light scattering and black—low angle light scattering).

Considering the different chemical structure of chitosan and gelatin, molecular weight distribution analysis was performed on the polymer-MFs to better define the features of the functionalized starting polymers. Size exclusion chromatography (or HP-SEC-TDA) coupled with analysis on multi-detector systems to capture light scattering, refractive index, and viscosity were employed to characterize gelatin-MF and chitosan-MF compared to the starting untreated counterparts. HP-SEC-TDA was used extensively to obtain molecular weight distribution of polymers and proteins; this method does not require any chromatographic calibration and is suitable to analyze polymers of different natures and weights. The solvents used in the analysis range from near-physiological to very harsh conditions, including high salts and/or strong surfactants concentration. The solvent systems induce complete denaturation or loss of higher orders protein structure of gelatin. In this way, good separations are obtained, and accurate molecular weight information can be acquired. Chromatographic profiles of gelatin and gelatin-MF are visible in Figure 1a,b, with a large bell-shape chromatographic peak, with a quite high polydispersion index value. 

The refractive index elution profiles of the MF derivates (Figure 1b,d) show the enlargement of the profile in the low-molecular-weight chains side respect to gelatin and chitosan (Figure 1a,c), suggesting a possible interaction with the stationary phase. Anyway, this phenomenon does not influence the molecular weight distribution evaluation. 

The average results of two analyses for gelatin and gelatin-MF, in terms of Mw (weight average molecular weight), Mn (number average molecular weight), dispersity D (expressed as Mw/Mn ratio), and intrinsic viscosity ((η), dL/g) values are reported in Table 1. The obtained results reveal high variations in molecular weight distribution and viscosity between the gelatin samples analyzed. 

The obtained results reveal no significant variations in molecular weight values and viscosity between the gelatin and gelatin-MF samples analyzed. As expected, the derivatization of amino groups of lysine to N-methyl-(5-methylfurfuyl), after reductive amination with 5-methylfurfural, does not significatively change the molecular weight values.

Chromatographic profiles of chitosan and chitosan-MF are shown in Figure 1c,d: in both cases the sample is eluted between 11 and 18 mL, with a large asymmetric bell-shape chromatographic peak, indicating a quite high polydispersion index value. Here, the comparable values obtained confirm the maintenance of starting chitosan structure and molecular weight after the reaction with 5-methylfurfural.

The average results of two analyses, in terms of Mw, are reported in Table 2. 

### 3.2. UV and FT-IR Analysis 

**Figure 2:**
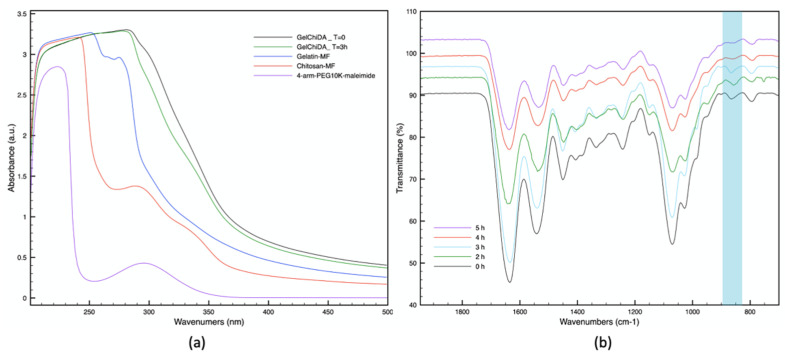
(**a**) UV spectra of cross-linking reaction in real time and (**b**) comparison of FT-IR spectra at different time points.

UV analysis allows to monitor the GelChiDA hybrid hydrogel formation by measuring the maleimide peak at 293 nm from T0 to T3 h [45]. This absorbance change is associated to the maleimide peak decrease due to Diels-Alder reaction between furan and maleimide moieties. This absorbance change is associated to the maleimide peak decrease due to Diels-Alder reaction between furan and maleimide moieties, leading to the cross-linked product formation, as shown in Figure 2a [30,45,46,47].

Figure 2b shows the evolution of IR spectra versus time of the reaction gelatin-MF and chitosan-MF with 4-arm-PEG10K-maleimide in PBS pH 7.4. We focused our attention on the signal at 862 cm^–1^, attributed to C-H vibration of double bond in furan ring. The peak shows a decrease in intensity over the time during the reaction, meaning the formation of the Diels-Alder adduct and the consequent cross-linking [48,49].

### 3.3. ^1^H-NMR Characterization 

**Figure 3:**
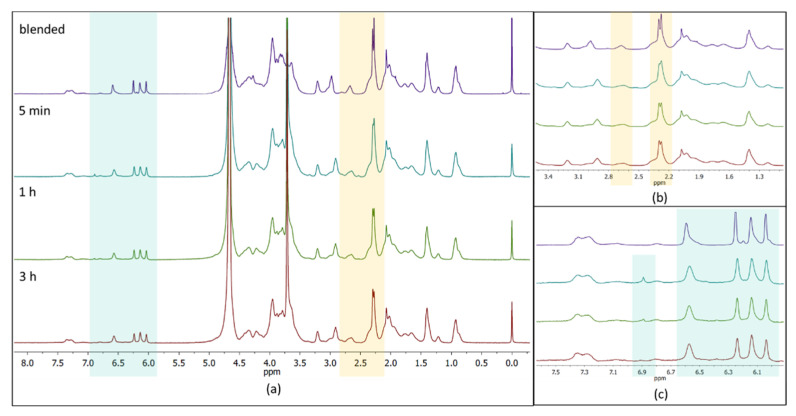
Comparison between ^1^H NMR spectrum of blended polymers and real-time gel formation at 5 min, 1 h, and 3 h (**a**) with a focus on the area between 1–3.5 (**b**) and 6–7.5 ppm (**c**).

Chitosan-MF and gelatin-MF were characterized by ^1^H-NMR analysis to evaluate the functionalization degree and the peaks assignment [50,51,52,53] (Figures S1 and S5). In-depth NMR characterization via ^13^C-NMR, HSQC, and gHMBC analysis of starting materials can be found in the Supplementary Materials. Particular attention has been devoted to the reacting groups. Each peak of furan of both chitosan-MF and gelatin-MF have been identified in both the ^1^H and ^13^C spectra comparing HSQC and gHMBC analysis, for short- and long-range correlations. Furthermore, peaks of interest have been characterized in order to determine the proper functionalization of the biopolymers prior to the hydrogel formulation. The peaks identified in both polymers have then been employed to follow the appropriate signals during the real-time NMR analysis in solution. Spectral variations, such as reduction in peak intensities, line broadening, and chemical shift changes, are all aspects monitored to assess the degree of cross-linking reaction and to determinate the reaction kinetics. The reaction conditions were replicated using deuterated PBS at pH 7.4 and 310 K in an NMR tube to follow the cross-linking kinetics until completion. Different representative spectra intervals have been considered. Figure 3a shows the spectra of the hydrogel formation in real time. 

**Figure 4:**
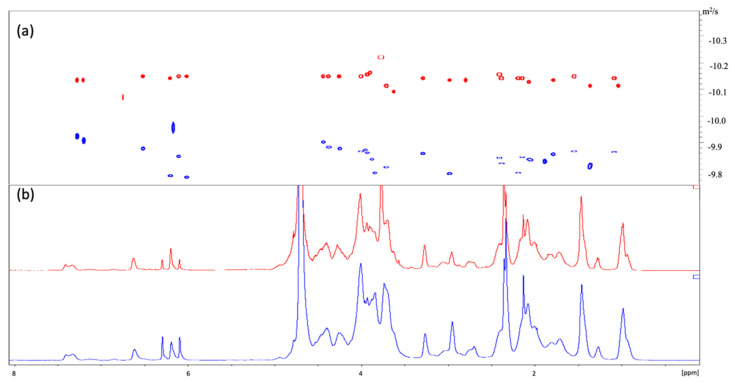
(**a**) Diffusion ordered spectroscopy NMR (DOSY) and (**b**) 1H spectra of blend (blue) and hydrogel (red) at the end of kinetics.

The kinetics was monitored through the acquisition of several ^1^H solution NMR spectra as reported in the Materials and Method section. After mixing the two polymers, the ^1^H-NMR spectrum shows the characteristic signal of 4-arm-PEG10K-Maleimide at 6.9 ppm [54] (Figure 3a,c). Observing the comparison of different timepoints of the kinetics, the signal of the double bond of maleimide decreases and finally, disappears after 3 h suggesting the completion of the reaction [55,56]. Simultaneously, the MF peaks between 6.0 and 6.6 ppm decrease, indicating a partial conversion of furan groups into the adduct with respect to the stoichiometry of the reaction (Figure S9). Moreover, the peak influenced by the signal of H-2 of chitosan-MF at 2.6 ppm (CHN) shifts by experiencing a change in the neighboring chemical environment, coherently with the reaction advance. Because of the moderate intensity of the new peak and the very slight shift downfield, such change in that region of the spectrum is only observed by integrating different areas of the overlapping signals, as shown in Figure S9. The peaks at 2.2 and 2.3 ppm, assigned to the methyl groups on methylfuran moieties of both polymers, undergo a shift as highlighted in the Figure 3b. To further analyze the cross-linked GelChiDA hybrid hydrogel and discriminate it with respect to the blended components, pulsed field gradient (PFG) diffusion ordered spectroscopy (DOSY) has been performed. The DOSY experiment is represented in a 2D spectrum in Figure 4, with chemical shift along one axis and the diffusion coefficients along the other. As shown in Figure 4a, just one population is detectable for the GelChiDA hydrogel (red line), whereas the blended chitosan-MF and gelatin-MF polymers (blue line) are characterized by different diffusive fronts.

In the blend sample, the diffusion values are lower if compared to the cross-linked hydrogel and the results showed different diffusive fronts, in particular for the peaks in the aromatic region between 7 and 6 ppm, rows with different gradient diffusion are detectable showing the signal at higher diffusion related to the furan protons of chitosan (6.3—6.1 ppm), and the signal at lower diffusion related to the furan protons of gelatin (6.2 ppm). Furthermore, the different mobility of the two blended polymers is detectable in the anomeric region between 4.5 and 3.0 ppm due to the different molecular weights of free chitosan and gelatin. The outcome is a different of diffusion coefficients of the blended sample, that is no more observed in the crosslinked hydrogel.

This result suggests that unreacted free polymers are absent in the solution, and the cycloaddition reaction with 4-arm-PEG10K-maleimide involved every available polymer chains. The comparison of the diffusion coefficients of the starting polymer and of the polymer treated with cross-linking agents facilitates the verification of hydrogel formation [57]. To distinguish different species, apparent self-diffusion coefficients (D) were determined. In the gelatin-chitosan hydrogel, the average value of diffusion coefficient (D) is in the order of 10–12 m^2^/s, which is significantly reduced compared to the values of chitosan-MF and gelatin-MF or the blend (in the order of 1.10–11 m^2^/s). The D value decreases because of the covalent bonds between the two polymers, which involve stronger linkages, stronger interactions, and then lower diffusion, as shown in Figure 4. Moreover, the figure shows how blended chitosan-MF and gelatin-MF have different diffusion coefficients (in blue), with respect to the cross-linked hydrogel (in red).

### 3.4. Thermogravimetric Analysis (TGA)

**Figure 5:**
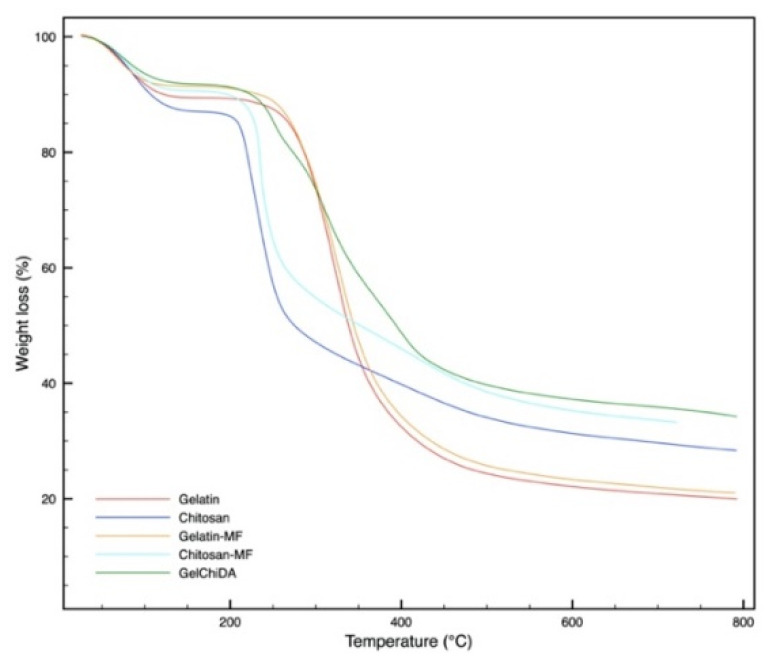
TGA curves of functionalized and unfunctionalized gelatin and chitosan and gelatin-chitosan cross-linked hydrogel.

TGA results of pure and functionalized gelatin and chitosan and GelChiDA are shown in Figure 5. The gelatin curves exhibit two thermal decomposition steps. The first step occurs up to 363 K due to moisture weight loss. The second one takes place in the temperature range of 473–773 K, attributed to the complete breakdown of protein chains. Temperature onset and maximum degradation temperatures (Tonset and Tmax) are observed at 493 and 573 K, respectively. In TGA curves of chitosan, besides the weight loss up to 393 K due to the adsorbed water, the main thermal degradation occurs in one step and starts at 468 K that is initiated by the random chain break and deacetylation. The maximum degradation temperature is reached at 508 K [58]. In both functionalized chitosan and gelatin, TGA analyses show a slight improvement of thermal properties as reported in Table 3. 

TGA curves for GelChiDA are quite similar except that thermal stability is improved by cross-linking (Figure 5). The second degradation stage of hybrid hydrogel takes place at higher temperature than the corresponding stage of pure chitosan but at lower temperature compared to gelatin, indicating that the composite has in-between trend [59]. As the graph points out, the hybrid biomaterial has a lower % weight loss at high temperatures. In other words, the product has higher thermal stability than starting materials [60].

## 4. Discussion

3D bioprinting of hybrid hydrogels is currently a hot topic and represent a valid manufacturing methodology to implement and maximize the translation of 3D tissue models for drug screening and tissue engineering applications [61,62]. GelChiDA, a hydrogel that we developed by cross-linking gelatin and chitosan, satisfies multiple critical requirements, including cell compatibility, enhanced printability, structural and chemical stability, appealing kinetics, and rheological properties. Such a complex platform has various advantages over traditional 2D cell cultures, providing an ECM-mimicking environment with better control over cell fate and signaling properties, and thus is highly promising for translational applications in biomedicine and tissue engineering [63]. It has application in translational research, however, requires in-depth characterization of the chemical and structural properties of the final 3D printable construct. A set of techniques has been proposed, achieving an in-depth and complete characterization of both the starting materials and the final construct, including the cross-linking reaction kinetics. Chemical and physical properties have been investigated employing HP-SEC-TDA, UV, FT-IR, NMR, and TGA. The proposed analytical strategy guarantees a relatively rapid but complete overview over the critical properties of such complex biomaterials and can be, in principle, applied to all the constructs of this nature, for both the optimization and tuning of the properties of a new material and the quality control of well-established platforms. Furthermore, the completeness and depth of characterization may provide more and useful details depending on the hydrogel investigated. For example, some of the proposed techniques such as DOSY allow to verify the absence of unreacted and undesirable by-products, which can potentially compromise the biocompatibility of the hydrogels. 

## Figures and Tables

**Table 1:** Main results of the high-performance size exclusion chromatography on line with a triple detector array (HP-SEC-TDA) analysis for the gelatin and gelatin-methylfuran (MF) samples.

Sample	Mw kDa	Mn kDa	D (Mw/Mn)	(η) dL/g
Gelatin	210	115	1.9	0.51
Gelatin-MF	196	140	1.4	0.46

**Table 2:** Main results of the HP-SEC-TDA analysis for the chitosan and chitosan-MF samples.

Sample	Mw kDa	Mn kDa	D (Mw/Mn)	(μ) dL/g
Chitosan	47.5	28.7	1.7	1.5
Chitosan-MF	57.0	30.0	1.9	1.7

**Table 3:** TGA analysis of the starting and functionalized polymers and the hydrogel.

Sample	Water Desorption			Degradation		
	Peak (K)	Range (K)	Weight Loss (%)	Peak (K)	Range (K)	Weight Loss (%)
Gelatin	348	300–429	10.6	592	457–879	67.4
Gelatin-MF	340	300–421	8.6	597	443–877	68.1
Chitosan	361	300–428	12.9	499	453–634	44.7
Chitosan-MF	349	300–423	9.3	508	434–616	40
GelChiDa	348	300–429	8.2	582	440–923	55.3

## Data Availability

Not applicable.

## Supplementary Materials

The following are available online at https://www.mdpi.com/article/10.3390/biom11050683/s1, Figure S1: ^1^H-NMR—chitosan-MF, Figure S2: ^13^C NMR—chitosan-MF, Figure S3: HSCQ—chitosan-MF, Figure S4: gHMBC—chitosan-MF, Figure S5: ^1^H-NMR—gelatin-MF, Figure S6: ^13^C-NMR—gelatin-MF, Figure S7: gHMBC—gelatin-MF, Figure S8: ^1^H-NMR—4-arm-PEG10K-Maleimide, Figure S9: ^1^H-NMR—integrals of hydrogel at different timepoints.

## Abbreviations

ECM	Extracellular matrix
MF	Methylfuran
GelChiDA	Gelatin-chitosan Diels Alder hybrid hydrogel
HP-SEC-TDA	High-performance size exclusion chromatography on line with a triple detector array
NMR	Nuclear magnetic resonance
DOSY	Diffusion order spectroscopy NMR
FTIR-ATR	Fourier-transform infrared spectroscopy-attenuated total reflection
UV-VIS	Ultraviolet–visible spectroscopy
TGA	Thermogravimetric analysis
