# Supplementary material for: Combined Analytical Approaches to Standardize and Characterize Biomaterials Formulations: Application to Chitosan-Gelatin Cross-Linked Hydrogels
Source: Biomolecules. 2021 May 1;11(5):683. doi: 10.3390/biom11050683 (PMC8147276; doi:10.3390/biom11050683)
Supplement: Supplementary file 1 [file biomolecules-11-00683-s001.zip › biomolecules-1202004-supplementary.pdf]

biomolecules

Supplementary Materials

Combined analytical approaches to standardize and characterize biomaterials formulations: application to Chitosan-Gelatin Crosslinked Hydrogels

Sofia Magli[1,2], Lorenzo Rossi[1,2], Cesare Consentino[4], Sabrina Bertini[4], Francesco Nicotra[1,2], Laura Russo[1,2,3*]

[1] Department of Biotechnology and Biosciences, University of Milano-Bicocca, Milan, Italy.
[2] BioNanoMedicine Center, University of Milano-Bicocca, Milan, Italy.
[3] CÚRAM SFI Research Centre for Medical Devices, National University of Ireland Galway, Galway, Ireland
[4] G. Ronzoni Institute for Chemical and Biochemical Research, Milan.
* Correspondence: laura.russo@unimib.it ; Tel. +39-0264483462 (L.R.)

Citation: Lastname, F.; Lastname, F.; Lastname, F. Title. *Biomolecules* **2021**, *11*, 683. https://doi.org/10.3390/biom11050683

Academic Editor: Firstname Lastname
Received: date
Accepted: date
Published: 1 May 2021

Publisher's Note: MDPI stays neutral with regard to jurisdictional claims in published maps and institutional affiliations.

Copyright: © 2021 by the authors. Licensee MDPI, Basel, Switzerland. This article is an open access article distributed under the terms and conditions of the Creative Commons Attribution (CC BY) license (http://creativecommons.org/licenses/by/4.0/).

Figure S1. ^{1}H-NMR – chitosan-MF

^{1}H NMR (deuterated PBS pH 7,4 with 0.05% wt 3-(trimethylsilyl)propionic-2,2,3,3-d$_4$ acid sodium salt, 310K, 400 MHz): δ_H 6.2 (0.20 H, s), 6.0 (0.20 H, s), 4-3.6 (5.55 H,m), 2.9 (0.65 H, s), 2.6 (0.20 H, s), 2.2 (0.60 H, s), 2.0 (0.45 H, s)

By comparison between the integrals of the peaks of H-8 (2.2 ppm), H-10 (6.2 ppm) and H-11 (6.0 ppm) of methylfuran, H-2b (2.6 ppm) on the functionalized monomer of chitosan and H-2a (2.9 ppm) of the unreacted monomers, we estimated the degree of functionalization as 20%.

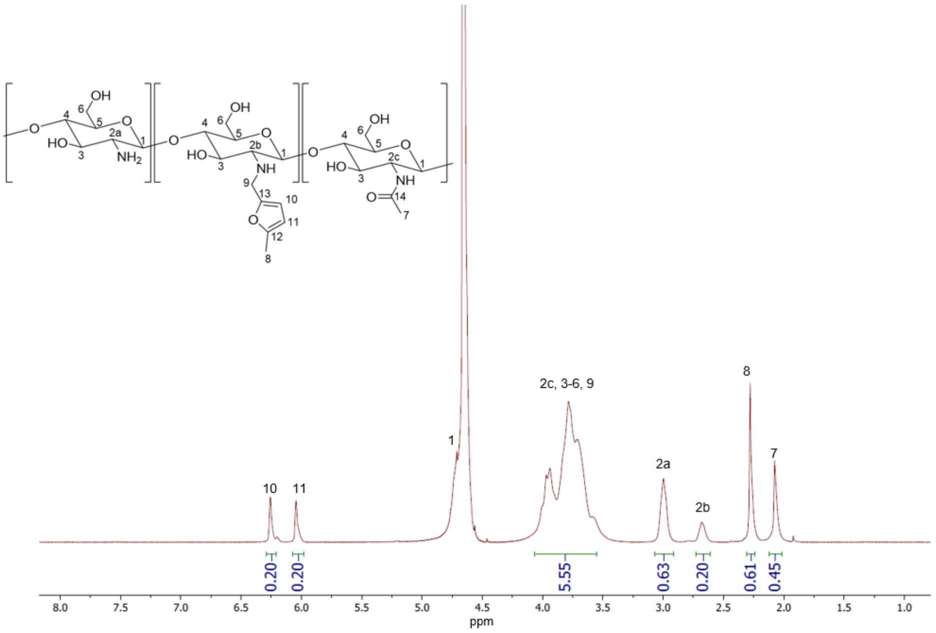

Figure S1. ^{1}H NMR spectrum of chitosan-MF.

Figure S2. ^{13}C NMR – chitosan-MF

^{13}C NMR (deuterated water, 310K, 400 MHz): δ_C 174.6, 152.7, 150.8, 109.0, 106.0, 102.2-100.5 (m), 77.5-71.6, 61.4, 60.2, 56.2, 55.4, 44.3, 22.2, 12.7.

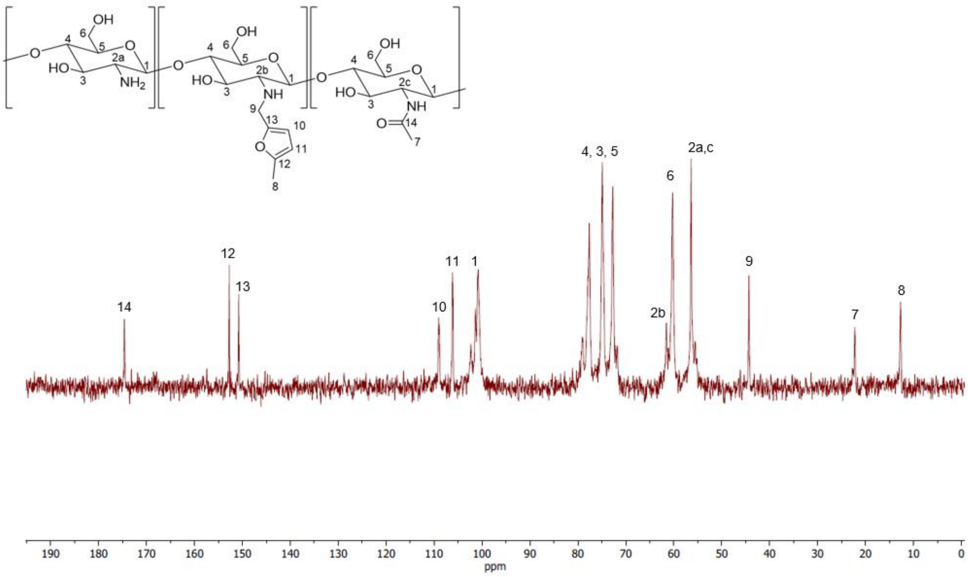

Figure S2. ^{13}C NMR spectrum of chitosan-MF.

Figure S3. HSCQ – chitosan-MF

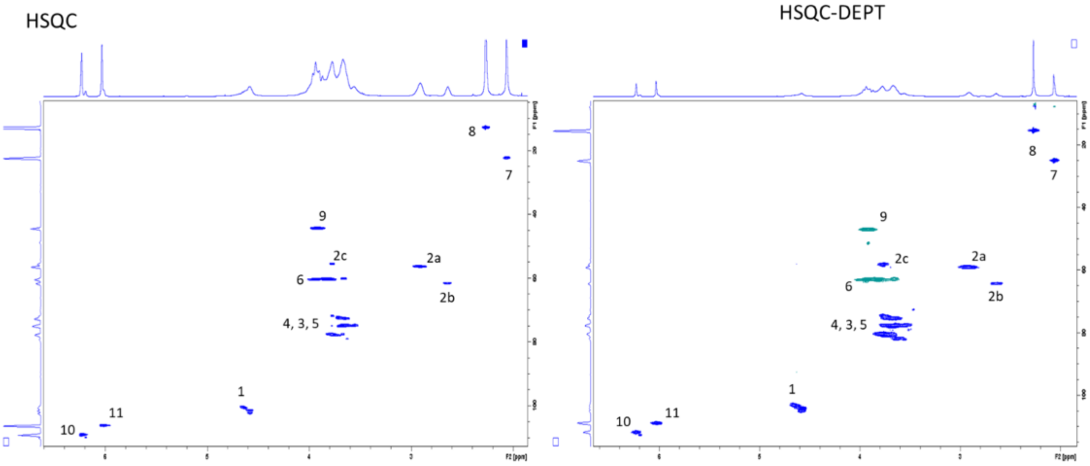

Figure S3. ^{1}H,^{13}C-HSQC/HSQC-DEPT spectra of chitosan-MF.

HSQC data were acquired in the phase-sensitive mode by using time proportional phase incrementation (TPPI) or States-TPPI experiment. Spectra were collected as

1024/320 TD experiments each with 2k complex data points and 20 scans over a spectral width of 5 kH.

Figure 4. gHMBC – chitosan-MF

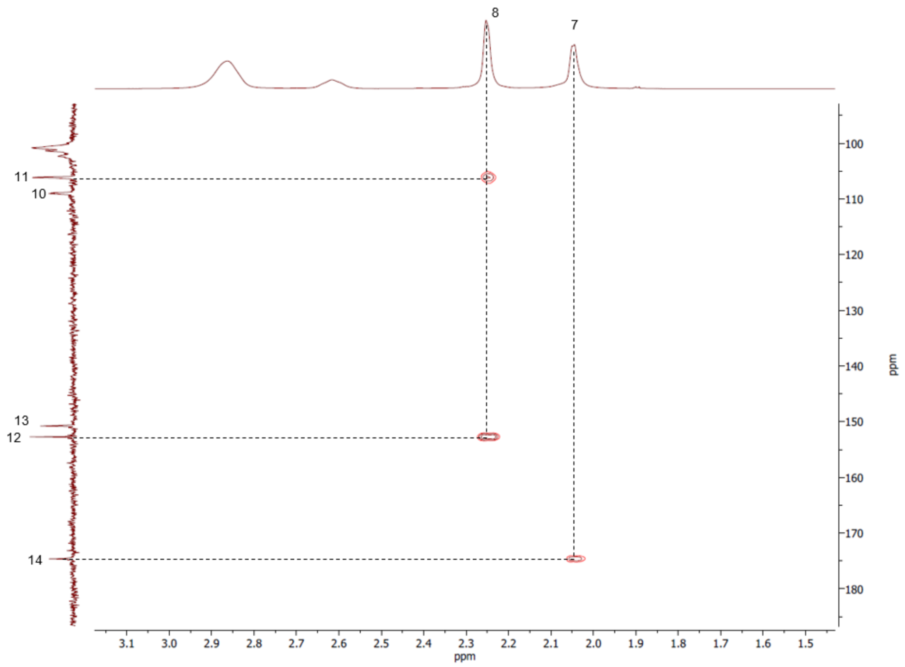

Figure S4. gHMBC spectrum of chitosan-MF.

gHMBC spectrum was obtained with 200 increments of 512 transients each and relaxation delay of 2 s. The gHMBC experiment was optimized for long-range J-coupling ^{1}H-^{13}C of 8 Hz. In particular, H-8 (2.2 ppm) correlates with C-11 (106.0 ppm) and C-12 (152.7 ppm), while H-7 (2.0 ppm) correlates with C-14 (174.6 ppm).

Figure S5. ^{1}H-NMR – gelatin-MF

^{1}H NMR (deuterated PBS pH 7,4 with 0.05% wt 3-(trimethylsilyl)propionic-2,2,3,3-d4 acid sodium salt, 310K, 400 MHz): δ_H 6.5 (s), 6.1 (s), 4.2 (s), 2.3 (s).

By comparison between the integral of the peaks of H-1 (6.5 ppm) and H-2 (6.1 ppm) in methylfuran and the peak of the reference, we calculated the amount of methylfuran. In particular, we estimated $6.29 \cdot 10^{-4}$ mmol of methylfuran per mg of gelatin-MF. Considering the weighted average of aminoacids' molecular weight, we estimated the degree of functionalization as 12,7%. The signal of the methyl moiety at 2.3 ppm has not been considered since it is influenced by the signals of aminoacidic chains in gelatin. Furthermore, by comparison with the spectrum of gelatin (**B**), the disappearance of the peak at 3.0 ppm, corresponding to the protons on the chain of lysine, is observable.

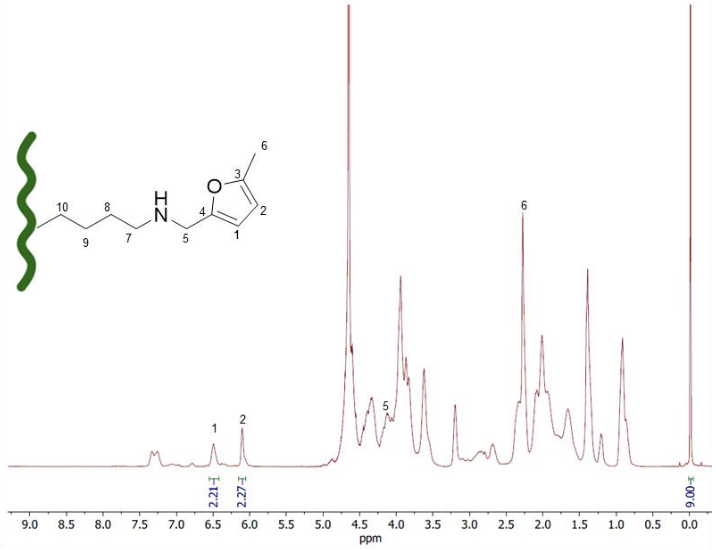

(a)

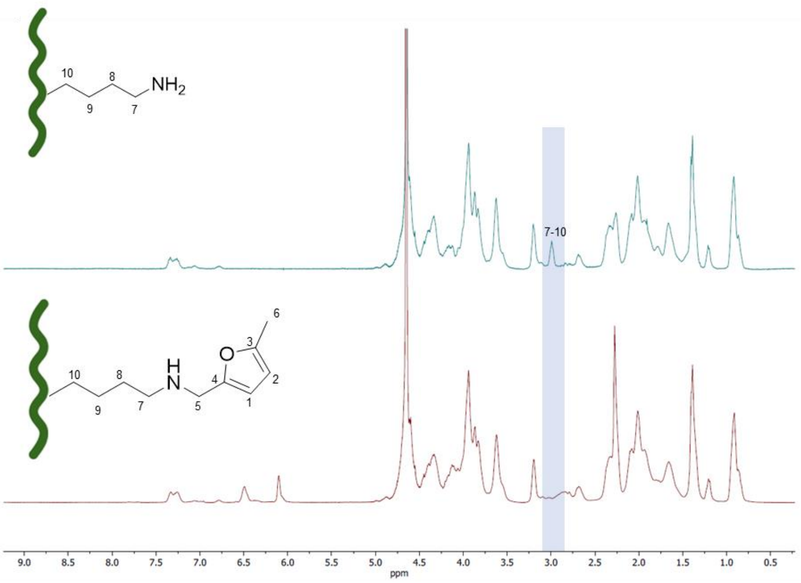

(b)

Figure S5. **A)** ^{1}H NMR spectrum of gelatin-MF. **B)** comparison between ^{1}H NMR spectra of gelatin and gelatin-MF.

Figure S6. ^{13}C-NMR – gelatin-MF

^{13}C NMR (deuterated water, 310K, 400 MHz): δ_C 181-168, 156.8, 155.5, 141.5, 129.4-128.7, 115.7, 107.1, 70.0, 67.1-14.9, 12.9

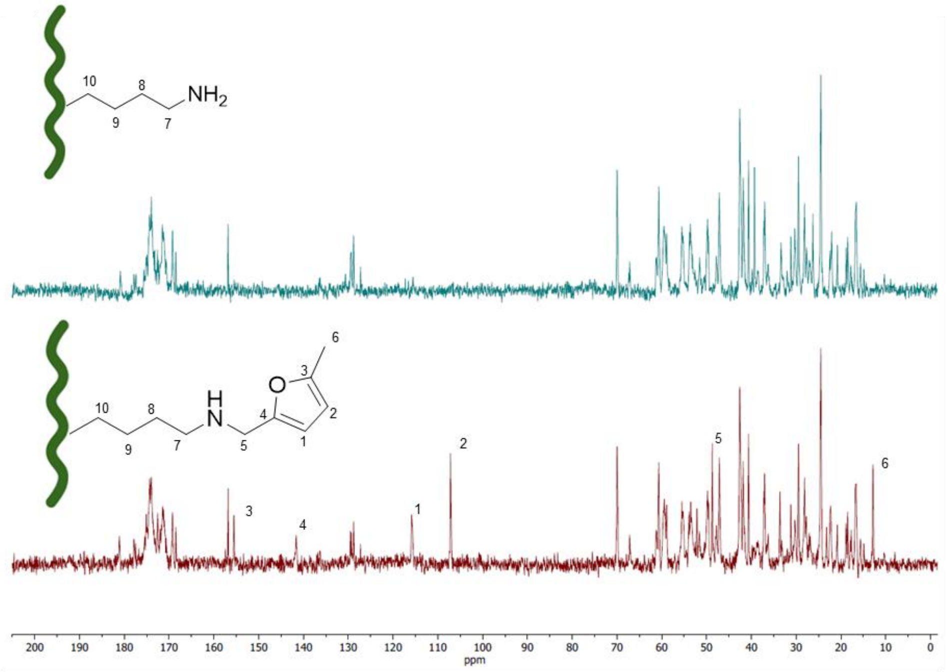

(a)

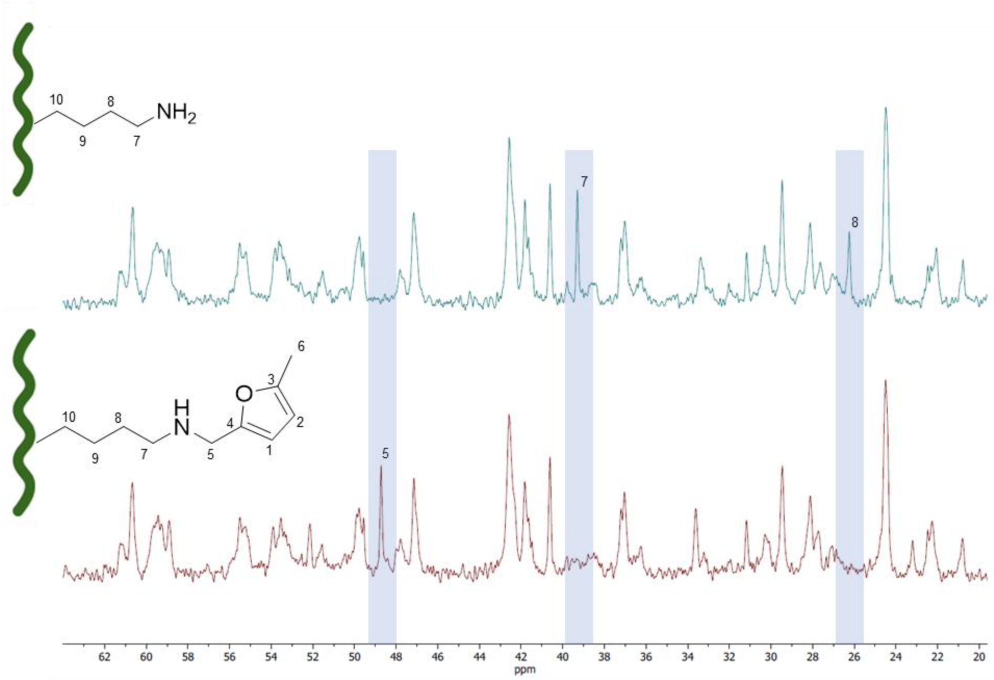

(b)

Figure S6. A) comparison between ^{13}C NMR spectra of gelatin and gelatin-MF. **B)** focus on the area between 62 and 20 ppm.

Figure S7. gHMBC – gelatin-MF

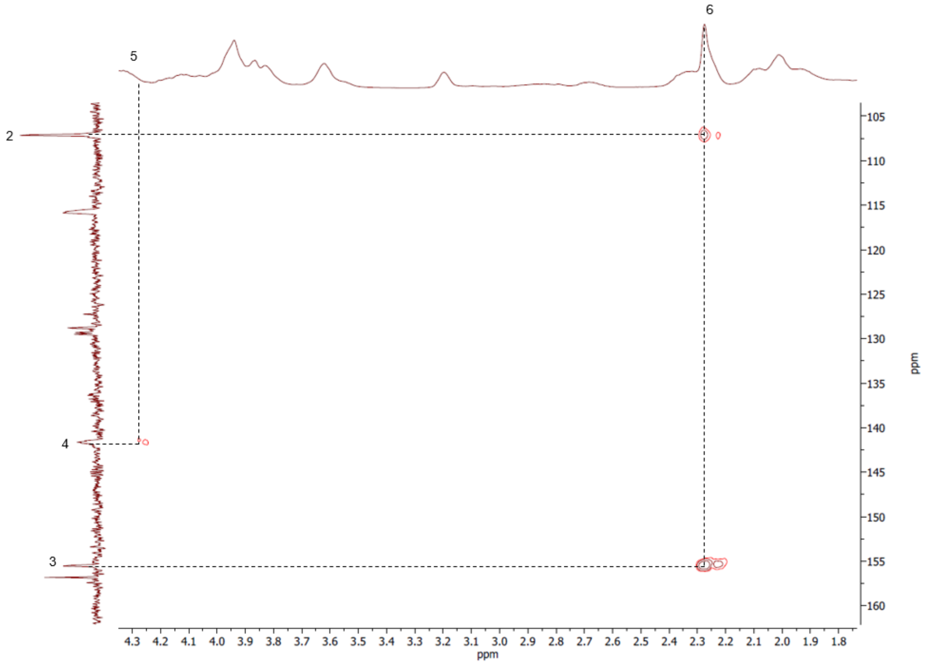

Figure S7. gHMBC spectrum of gelatin-MF.

gHMBC spectrum was obtained with 200 increments of 512 transients each and relaxation delay of 2 s. The gHMBC experiment was optimized for long-range J-coupling ^{1}H-^{13}C of 8 Hz. In particular, H-6 (2.3 ppm) correlates with C-2 (107.1 ppm) and C-3 (155.5 ppm), while H-5 (4.3 ppm) correlates with C-4 (141.5 ppm).

Figure S8. ^{1}H-NMR - 4arm-PEG10K-Maleimide

^{1}H NMR (deuterated PBS pH 7,4 with 0.05% wt 3-(trimethylsilyl)propionic-2,2,3,3-d_4 acid sodium salt, 310K, 400 MHz): δ_H 6.90 ppm (8H, s), 3.71 ppm (PEG backbone, s)

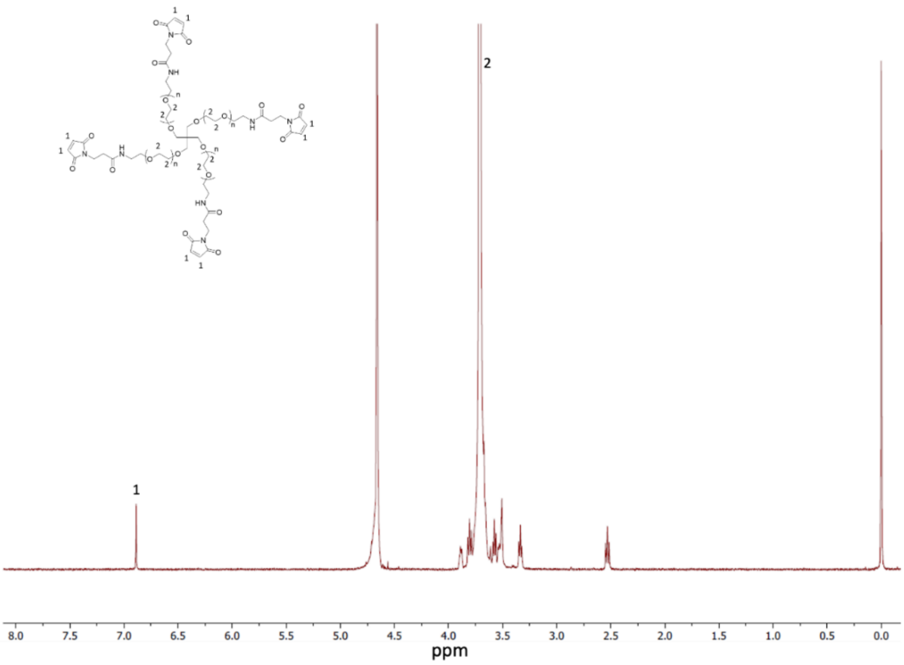

Figure S8. ^{1}H NMR spectrum of 4arm-PEG10K-Maleimide.

Figure S9. ^{1}H-NMR – Integrals of hydrogel at different timepoints

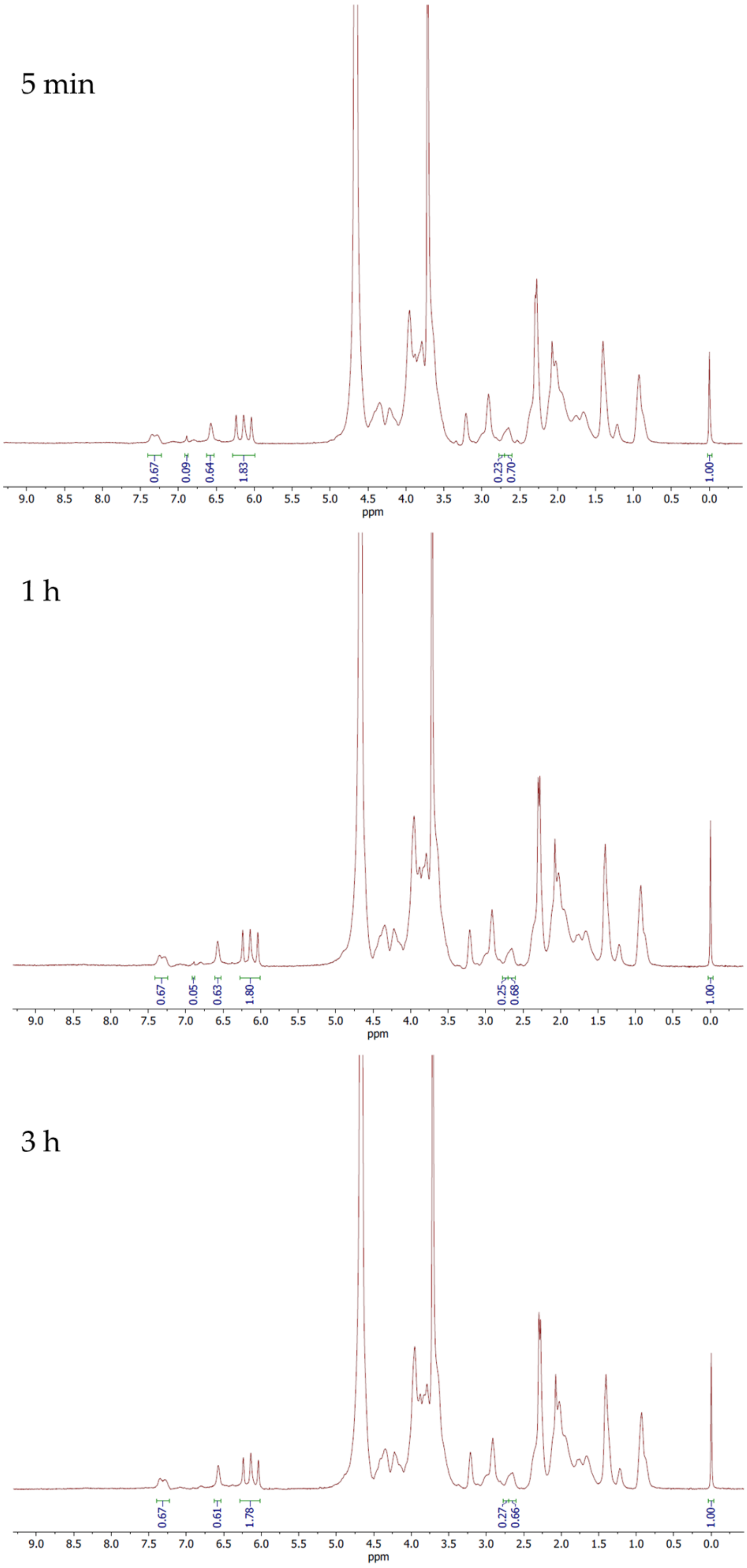

Figure S9. ^{1}H NMR spectrum of hydrogel at 5 minutes and 3 hours.
